# Peroxygenase activity of cytochrome *c* peroxidase and three apolar distal heme pocket mutants: hydroxylation of 1-methoxynaphthalene

**DOI:** 10.1186/1471-2091-14-19

**Published:** 2013-07-30

**Authors:** James E Erman, Heather Kilheeney, Anil K Bidwai, Caitlan E Ayala, Lidia B Vitello

**Affiliations:** 1 Department of Chemistry and Biochemistry, Northern Illinois University, DeKalb, IL 60115, USA

**Keywords:** Cytochrome *c* peroxidase, Peroxygenase activity, Heme pocket mutants, 1-methoxynaphthalene

## Abstract

**Background:**

The cytochrome P450s are monooxygenases that insert oxygen functionalities into a wide variety of organic substrates with high selectivity. There is interest in developing efficient catalysts based on the “peroxide shunt” pathway in the cytochrome P450s, which uses H_2_O_2_ in place of O_2_/NADPH as the oxygenation agent. We report on our initial studies using cytochrome *c* peroxidase (CcP) as a platform to develop specific “peroxygenation” catalysts.

**Results:**

The peroxygenase activity of CcP was investigated using 1-methoxynaphthalene as substrate. 1-Methoxynaphthalene hydroxylation was monitored using Russig’s blue formation at standard reaction conditions of 0.50 mM 1-methoxynaphthalene, 1.00 mM H_2_O_2_, pH 7.0_,_ 25°C. Wild-type CcP catalyzes the hydroxylation of 1-methoxynaphthalene with a turnover number of 0.0044 ± 0.0001 min^-1^. Three apolar distal heme pocket mutants of CcP were designed to enhance binding of 1-methoxynaphthalene near the heme, constructed, and tested for hydroxylation activity. The highest activity was observed for CcP(triAla), a triple mutant with Arg48, Trp51, and His52 simultaneously mutated to alanine residues. The turnover number of CcP(triAla) is 0.150 ± 0.008 min^-1^, 34-fold greater than wild-type CcP and comparable to the naphthalene hydroxylation activity of rat liver microsomal cytochrome P450. While wild-type CcP is very stable to oxidative degradation by excess hydrogen peroxide, CcP(triAla) is inactivated within four cycles of the peroxygenase reaction.

**Conclusions:**

Protein engineering of CcP can increase the rate of peroxygenation of apolar substrates but the initial constructs are more susceptible to oxidative degradation than wild-type enzyme. Further developments will require constructs with increased rates and selectivity while maintaining the stability of wild-type CcP toward oxidative degradation by hydrogen peroxide.

## Background

The cytochrome P450s form a large class of heme enzymes that catalyze hydroxylation or epoxidation of organic substrates (S) using molecular oxygen, Equation 1 [1-5]. During the

(1) $$\begin{array}{l} S+O_{2}+\mathrm{NADPH}+H^{+}\to \mathrm{SO}+\mathrm{NAD}P^{+}+H_{2}O\\ \;P450 \end{array}$$

catalytic cycle, molecular oxygen binds to the ferrous heme iron. Electron transfer from NADPH generates an enzyme intermediate called Compound I, which retains one of the oxygen atoms from O_2_ as a ferryl, porphyrin-radical species [6]. The oxygen atom from the ferryl heme is transferred to the organic substrate generating the oxygenated product. The rate of cytochrome P450-catalyzed reactions are generally slow with liver microsomal P450s having hydroxylation rates on the order of 1 min^-1^ while bacterial cytochrome P450s tend to have faster rates [2,7,8].

Cytochrome P450s have been used for synthetic purposes to insert oxygen functionalities with high selectivity into a wide variety of organic substrates [7,9]. However, they are not ideal synthetic catalysts. Cytochrome P450 requires an expensive cofactor in NADPH, generally has a low turnover rate, and is susceptible to oxidative degradation during the catalytic cycle. One of the first strategies to improve the P450s as synthetic catalysts involved elimination of the requirement for NADPH. Cytochrome P450 can react with H_2_O_2_ to oxygenate substrates in a pathway called the “peroxide shunt” Equation 2 [9]. The peroxide shunt involves H_2_O_2_ reacting

(2) $$\begin{array}{l} S+H_{2}O_{2}\to \mathrm{SO}+H_{2}O\\ \;P450 \end{array}$$

with the Fe(III) heme to generate Compound I. The peroxide shunt pathway is generally slower than the monooxygenase pathway and the enzyme is subject to oxidative degradation by the H_2_O_2_. Protein engineering approaches have been used to enhance the “peroxygenase” activity of the cytochrome P450s [10] as well as to incorporate peroxygenase activity into other heme proteins such as myoglobin and the peroxidases [11,12]. Recently, naturally occurring peroxygenases with high rates of activity have been discovered [13,14].

The peroxidases are a natural platform to explore peroxygenation reactions since the peroxidases have evolved to react efficiently with hydrogen peroxide and many are quite stable to oxidative degradation in the presence of excess hydrogen peroxide. Miller *et al.*[15] have shown that cytochrome *c* peroxidase (CcP) and a CcP mutant, CcP(W51A), have low-levels of peroxygenase activity, catalyzing the epoxidation of styrene and styrene derivatives by H_2_O_2_. The turnover numbers for epoxidation of *trans-β-*methylstyrene and *cis-β-*methylstyrene are about two orders of magnitude slower than the cytochrome P450 monooxygenase reaction.

One factor for the low peroxygenase activity of CcP may be the low affinity for apolar substrates within the heme pocket. Mutating the distal heme pocket to increase the binding of apolar substrates should increase the peroxygenase activity of CcP. In this study we report on the CcP-catalyzed steady-state hydroxylation of 1-methoxynaphthalene by H_2_O_2_ using the Russig’s blue assay developed by Shoji *et al.*[12]. Three CcP mutants with apolar distal heme pockets were designed to increase the solubility of typical monooxygenase and peroxygenase substrates within the distal heme pocket of CcP. They were constructed by simultaneously replacing Arg48, Trp51, and His52 with either all alanines, CcP(triAla), all valines, CcP(triVal), or all leucines, CcP(triLeu). We have previously reported on the reaction of these mutants with hydrogen peroxide and cyanide [16]. As anticipated, the reaction with H_2_O_2_ is substantially reduced but the peroxygenase activity is increased by a factor of 34.

## Methods

### Proteins

Both authentic yeast cytochrome *c* peroxidase, yCcP, and a recombinant CcP with the exact amino acid sequence of yCcP, rCcP, were used in this study. Isolation and purification of CcP has been described previously [16,17]. The cloning, expression and purification of CcP mutants has been described [16,18-21]. The three CcP triple mutants constructed for this study are CcP(triAla) – (R48A/W51A/H52A), CcP(triVal) – (R48V/W51V/H52V), and CcP(triLeu) – (R48L/W51L/H52L).

### Other materials

Potassium phosphate salts, potassium acetate, and hydrogen peroxide (30%) were obtained from Fisher Scientific. Hydrogen peroxide solutions were standardized by titration with Ce(IV) as described previously [22]. 1-Methoxynaphthalene was obtained from Aldrich Chemical Company.

### Spectroscopic measurements and protein concentration determination

Spectra of protein solutions were determined using either a Varian/Cary Model 3E or a Hewlett Packard Model 8452A spectrophotometer. Protein concentrations were determined from the absorption spectra using the maximum extinction coefficients in the Soret region at pH 6: yCcP 98 mM^-1^ cm^-1^ at 408 nm, rCcP 101 mM^-1^ cm^-1^ at 408 nm,. CcP(triAla) 110 mM^-1^ cm^-1^ at 406 nm, CcP(triVal) 76 mM^-1^ cm^-1^ at 406 nm, and CcP(triLeu) 93 mM^-1^ cm^-1^ at 400 nm [16,17].

### Steady-state kinetic measurements of 1-methoxynaphthalene hydroxylation

**Figure 1:**
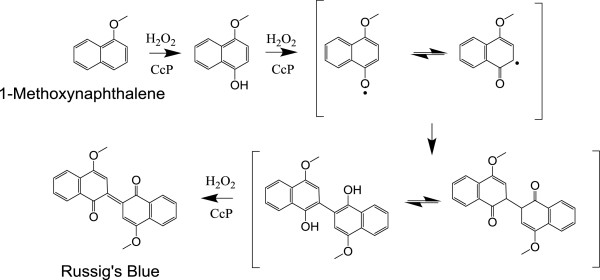
**Proposed reaction sequence for conversion of 1-methoxynaphthalene to Russig’s blue.** Adapted from reference [12].

1-Methoxynaphthalene hydroxylation was monitored by following formation of Russig’s blue at 610 nm [12]. A reaction mechanism for Russig’s blue formation is shown in Figure 1. The initial change in absorbance is converted to an initial velocity, *v*_*0*_, using Equation 3. The change

(3) $$v_{0}=2\left(\mathrm{dA}/dt\right)/\Delta \epsilon$$

in extinction coefficient, Δϵ, for Russig’s blue formation in aqueous solution is 1.45 × 10^4^ M^-1^ cm^-1^ at 610 nm ( in the Additional file 1: Figure SA.1). The factor of 2 in Equation 3 is required since two molecules of 1-methoxynaphthalene must be hydroxylated to produce one molecule of the product, Russig’s blue (Figure 1). Standard assay conditions were 0.50 mM 1-methoxynaphthalene, 1.0 mM hydrogen peroxide, enzyme as required (generally in the μM concentration range), pH 7.0, 0.10 M ionic strength potassium phosphate buffer, 25°C. The reaction was initiated by addition of hydrogen peroxide.

## Results

### Steady-state hydroxylation of 1-methoxynaphthalene by CcP and the apolar distal heme pocket mutants of CcP

**Figure 2:**
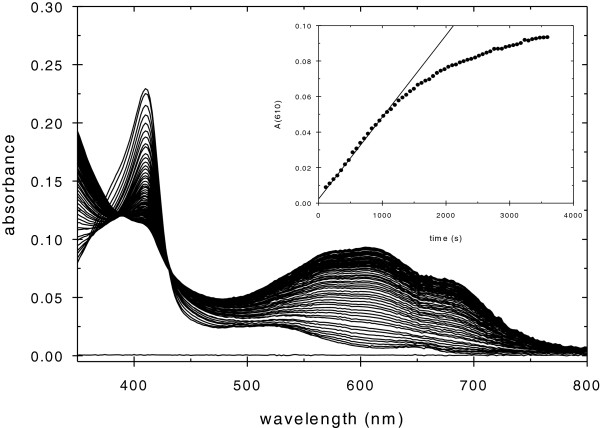
**CcP(triAla)-catalyzed oxidation of 1-methoxynaphthalene by hydrogen peroxide.** Spectra were collected every minute for one hour after addition of hydrogen peroxide. The large increase in absorbance between 450 and 800 nm is due to Russig’s blue formation. Inset – Increase in the absorbance at 610 nm as a function of time after hydrogen peroxide addition. Experimental Conditions: [CcP(triAla)] = 2.0 μM, [1-methoxynaphthalene] = 0.50 mM, [H_2_O_2_] = 1.00 mM, pH 7.0, 0.100 M ionic strength potassium phosphate buffer, 25°C.

**Figure 3:**
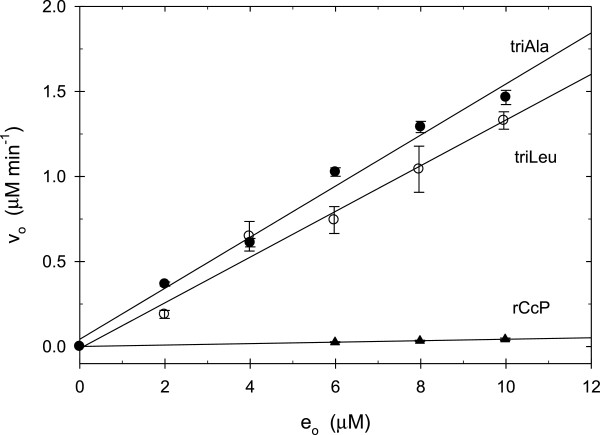
**Plots of initial velocity, v**_**0**_**, for 1-methoxynaphthalene hydroxylation as a function of initial enzyme concentration, e**_**0**_**. CcP(triAla) – solid circles, CcP(triLeu) – open circles, rCcP – solid triangles.** Experimental Conditions: [1-methoxynaphthalene] = 0.50 mM, [H_2_O_2_] = 1.00 mM, pH 7.0, 0.100 M ionic strength potassium phosphate buffer, 25°C.

We have utilized the Russig’s blue assay developed by Shoji and coworkers [12] to assess the 1-methoxynaphthalene hydroxylation activity of CcP and the three CcP mutants with apolar distal heme pockets, CcP(triAla), CcP(triLeu), and CcP(triVal). Figure 2 shows the CcP(triAla) catalyzed oxidation of 1-methoxynaphthalene by hydrogen peroxide at pH 7.0. There is a large increase in the absorbance between 500 and 800 nm during the reaction that is characteristic of Russig’s blue formation resulting from the hydroxylation of 1-methoxynaphthalene, Figure 1[12]. The inset of Figure 2 shows the increase in the absorbance at 610 nm as a function of time from which the initial velocity can be determined, Equation 3. The initial velocity increases linearly with increasing CcP(triAla) concentration, Figure 3, from which a turnover number of 0.150 ± 0.008 min^-1^ can be determined, Table 1.

**Table 1:** Peroxygenase activity of ccp and ccp mutants

**Enzyme**	**Substrate**	**Substrate concentration (mM)**	**pH**	**TN (min**^**-1**^**)**
rCcP	1-methoxynaphthalene	0.50	7.0	0.0044 ± 0.0001
CcP(triAla)	1-methoxynaphthalene	0.50	7.0	0.150 ± 0.008
CcP(triVal)	1-methoxynaphthalene	0.50	7.0	>0.137
CcP(triLeu)	1-methoxynaphthalene	0.50	7.0	0.134 ± 0.014

Spectral scans of the CcP(triLeu), CcP(triVal), and rCcP catalyzed Russig’s blue formation are similar to those for CcP(triAla) and are shown in the Additional file 1 associated with this manuscript. Initial velocities as a function of the CcP(triLeu) and rCcP concentration are shown in Figure 3, along with the CcP(triAla) data. The initial velocities increases linearly with increasing CcP(triLeu) and rCcP concentrations. Turnover numbers are included in Table 1. The initial velocity for the CcP(triVal)-catalyzed oxidation of 1-methoxynaphthalene is independent of the CcP(triVal) concentration between 5 and 10 μM and only a minimum value of 0.137 min^-1^ can be established for the CcP(triVal) turnover rate.

The rate of 1-methoxyhaphthalene hydroxylation catalyzed by CcP(triAla) was studied as functions of the 1-methoxynaphthalene and hydrogen peroxide concentrations. The initial velocity increases linearly with 1-methoxynaphthalene concentration and with the hydrogen peroxide concentrations (Additional file 1). Apparent second-order rate constants for the interaction of 1-methoxynaphthalene with CcP(triAla) Compound I and of hydrogen peroxide with CcP(triAla) are 1.5 ± 0.2 M^-1^ s^-1^ and 1.1 ± 0.1 M^-1^ s^-1^, respectively.

At the high concentrations of hydrogen peroxide used in the Russig’s blue assay, significant bleaching of the Soret absorption band occurs during the reaction, Figure 2, indicating heme destruction throughout the assay. This diminishes the catalytic effectiveness of CcP and its mutants. In a study of the hydroxylation of 1-metholxynaphthalene catalyzed by CcP(triAla), the formation of Russig’s blue was followed until cessation of the reaction. Using 10 μM CcP(triAla), 19.5 μM Russig’s blue was formed, corresponding to the hydroxylation of 39 μM 1-methoxynaphthalene.

### Screening of heme pocket mutants of CcP for 1-methoxynaphtalene hydroxylation activity

**Figure 4:**
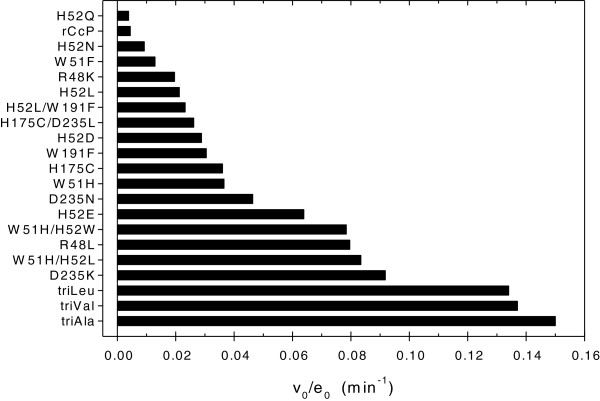
**Turnover rates, *v***_***0***_**/*e***_***0***_**, for 1-methoxynaphthalene hydroxylation by hydrogen peroxide catalyzed by rCcP and 17 CcP mutants.** Experimental Conditions: [1-methoxynaphthalene] = 0.50 mM, [H_2_O_2_] = 1.00 mM, pH 7.0, 0.100 M ionic strength potassium phosphate buffer, 25°C.

We screened all of the CcP heme pocket mutants that were available in our laboratory for 1-methoxynaphthalene activity to determine if any had unusually high peroxygenase activity or higher stability toward heme degradation during the peroxygenase reaction. The results are shown in Figure 4. Turnover numbers for the CcP mutants not specifically designed to bind apolar substrates ranged from 0.0038 ± 0.0007 min^-1^ for CcP(H52Q) to 0.092 ± 0.004 min^-1^ for CcP(D235K). Two CcP mutants designed to mimic the heme axial ligation of cytochrome P450, CcP(H175C) and CcP(H175C/D235L), have hydroxylation rates of 0.036 ± 0.001 and 0.026 ± 0.001 min^-1^, respectively. Turnover numbers (*v*_*0*_/*e*_*0*_) for all CcP mutants determined in this study are collected in the Additional file 1: Table SA.1. Heme degradation for all mutants is shown in Additional file 1: Figure SA.8. CcP(H52N) is most resistant to heme degradation during the peroxygenase reaction while CcP(W51H) is most susceptible.

## Discussion

Relatively few studies of naphthalene hydroxylation by the cytochrome P450s have been reported. Rat liver microsomes hydroxylate naphthalene with a monooxygenation turnover rate of 0.32 min^-1^[23] and cytochrome P450_cam_ hydroxylates naphthalene with a rate of 0.7 min^-1^[24]. Protein engineering of cytochrome P450_cam_ produced a Y96F mutant, which increases the rate of naphthalene hydroxylation to 100 min^-1^[24], the fastest reported for a cytochrome P450. Joo *et al.* have been using laboratory evolution techniques to enhance the peroxygenase activity of cytochrome P450_cam_[10]. Using a fluorescence technique to monitor naphthalene hydroxylation, Joo and colleagues screened approximately 200,000 random mutants of cytochrome P450_cam_ and were able to select a mutant that had 11-fold higher peroxygenase activity than did the wild-type enzyme although absolute rates were not reported.

Cytochrome P450_BSβ_ is a naturally-occurring peroxygenase, which hydroxylates fatty acids predominantly at the β position [14]. It has no monooxygenase activity and does not react with hydrogen peroxide in the absence of the fatty acid substrate. Myristic acid is the preferred substrate and the turnover rate is about 300 min^-1^. In the presence of a short-chain fatty acid, cytochrome P450_BSβ_ will catalyze the peroxygenation of a variety of nonnatural substrates such as styrene, ethylbenzene, and 1-methoxynaphthalene.

Shoji *et al.*[12] investigated the rates of peroxygenation of 1-methoxynaphthalene by three heme proteins, cytochrome P450_BSβ_, HRP, and metmyoglobin. HRP had no detectable activity, while sperm whale metmyoglobin has a turnover number of about 0.03 min^-1^. The H64A mutation of metmyoglobin increases the rate of Russig’s blue formation almost 800-fold to 23 min^-1^. Cytochrome P450_BSβ_ had a turnover number of 112 min^-1^ for 1-methoxynaphthalene hydroxylation.

In this study, we show that CcP has detectable hydroxylation activity with a turnover number of 0.0044 min^-1^, faster than that of HRP but slower than that of metMb and cytochrome P450_BSβ_. The three CcP mutants designed to bind non-polar substrates within the heme pocket, CcP(triAla), CcP(triVal), and CcP(triLeu), are about 30-fold more active than rCcP, however, none approached the activities of metMb(H64A) or cytochrome P450_BSβ_. Two CcP mutants, H175C and H175C/D235L, designed to simulate the heme ligation in cytochrome P450 have peroxygenase activities that are intermediate between those of rCcP and CcP(triAla).

One of the anticipated strengths of CcP as a platform to develop specific peroxygenase catalysts is the stability of the initial oxidized intermediate in the CcP/hydrogen peroxide reaction, CcP Compound I, and the stability with respect to oxidative degradation by excess hydrogen peroxide [25,26]. The half-life of CcP compound I is about 6 hours at pH 6 [25] and CcP can react with up to 10 equivalents of hydrogen peroxide in the absence of oxidizable substrates before significant reduction in catalytic activity occurs [26]. However, in the Russig’s blue assay, we saw rapid bleaching of the heme, Figure 2, and complete inactivation of CcP(triAla) within four catalytic cycles. In our screening process, we found that CcP(triAla) is one of the most susceptible mutants to heme degradation with a loss of 73% of the Soret absorbance during the Russig’s blue assay, in the Additional file 1: Figure SA.8. Interestingly, there is about a 10-fold variation in heme degradation during the Russig’s blue assay for the twenty CcP mutants screened in this study, Figure A8. CcP(H52N) is the most stable of the screened mutants with only 8% heme degradation while CcP(W51H) is the most susceptible mutant with 74% heme degradation. The degradation does not correlate with either the rate of compound I formation or with the rate of 1-methoxynaphthalene hydroxylation. It may be due to secondary oxidation of the hydroxylated product within the distal heme pocket, Figure 1, forming substrate-based radicals which, in turn, react with the heme causing the observed degradation.

## Conclusions

The 1-methoxynaphthalene hydroxylation activity of wild-type CcP can be increased 34-fold by making the distal heme pocket apolar. The 1-methoxynaphthalene hydroxylation rate of 0.150 ± 0.008 min^-1^ for CcP(triAla) is only a factor of two smaller than the naphthalene hydroxylation activity of rat liver microsomal cytochrome P450 but is still three orders of magnitude slower than that of the best naphthalene hydroxylation catalysts yet reported, the peroxygenation of 1-methoxynaphthalene by cytochrome P450_BSβ_ with a turnover number of 112 min^-1^[12] and the monooxygenase activity of cytochrome P450_CAM_(Y96F) with a turnover number of 100 min^-1^[24]. The increased rate of 1-methoxynaphthalene hydroxylation comes at the expense of decreased stability of CcP toward oxidative degradation in the presence of excess hydrogen peroxide. Further development of CcP mutants as peroxygenation catalysts will have to maintain the stability of the enzyme while increasing the rate of substrate turnover.

### Availability of supporting data

All supporting data are included in the additional file included with this article.

## Abbreviations

CcP: Generic abbreviation for cytochrome *c* peroxidase whatever the source; yCcP: Authentic yeast cytochrome *c* peroxidase isolated from *S. cervisiae*; rCcP: Recombinant cytochrome *c* peroxidase expressed in *E. coli*, which has an identical amino acid sequence to that of yCcP; CcP(triAla): Triple mutant of rCcP with R48A/W51A/H52A; CcP(triLeu): Triple mutant of rCcP with R48L/W51L/H52V; CcP(triVal): Triple mutant of rCcP with R48V/W51V/H52V.

## Competing interests

All authors declare that they have no competing interests.

## Authors’ contributions

JEE proposed and designed the experiments, carried out some of the Russig’s blue assays, and wrote the initial draft of the manuscript. LBV was responsible for constructing the triple mutants. AKB performed the initial characterization of the triple mutants. CEA helped develop the initial testing protocol including determining the spectra of Russig’s blue in aqueous solution. HK helped characterize the triple mutants and was involved in the Russig’s blue assays. All authors were involved in interpretation of the data, editing the manuscript, and all have read and approved the final manuscript.

## Supplementary Material

Additional file 1 **The additional file provided with this paper contains 8 figures and 1 table.Figure SA.1**. Spectrum of Russig’s blue in organic and aqueous solution. **Figure SA.2**, **Figure SA.2**, and **Figure SA.3**. Spectral scans CcP(triLeu)-, CcP(triVal)-, and rCcP-catalyzed formation of Russig’s blue, respectively. **Figure SA.5**. Dependence of the initial velocity for CcP(triAla)-catalyzed formation of Russig’s blue on the concentration of 1-methoxynaphthalene. **Figure SA.6**. Dependence of the initial velocity for CcP(triAla)-catalyzed formation of Russig’s blue on the concentration of hydrogen peroxide. **Figure SA.7**. Histogram of the peroxygenase activity of rCcP, 20 CcP mutants, and selected other heme proteins. **Figure SA.8**. Percent heme degradation during the peroxygenase reaction for the heme proteins included in **Figure SA.7**. **Table SA.1**. Peroxygenase activity of rCcP, 20 CcP mutants, and selected other heme proteins. Click here for file
