# Supplementary material for: Peroxygenase activity of cytochrome c peroxidase and three apolar distal heme pocket mutants: hydroxylation of 1-methoxynaphthalene
Source: BMC Biochem. 2013 Jul 30;14:19. doi: 10.1186/1471-2091-14-19 (PMC3733812; doi:10.1186/1471-2091-14-19)
Supplement: Additional file 1 — The additional file provided with this paper contains 8 figures and 1 table.Figure SA.1. Spectrum of Russig’s blue in organic and aqueous solution. Figure SA.2, Figure SA.2, and Figure SA.3. Spectral scans CcP(triLeu)-, CcP(triVal)-, and rCcP-catalyzed formation of Russig’s blue, respectively. Figure SA.5. Dependence of the initial velocity for CcP(triAla)-catalyzed formation of Russig’s blue on the concentration of 1-methoxynaphthalene. Figure SA.6. Dependence of the initial velocity for CcP(triAla)-catalyzed formation of Russig’s blue on the concentration of hydrogen peroxide. Figure SA.7. Histogram of the peroxygenase activity of rCcP, 20 CcP mutants, and selected other heme proteins. Figure SA.8. Percent heme degradation during the peroxygenase reaction for the heme proteins included in Figure SA.7. Table SA.1. Peroxygenase activity of rCcP, 20 CcP mutants, and selected other heme proteins. [file 1471-2091-14-19-S1.pdf]

Peroxygenase activity of cytochrome *c* peroxidase and three apolar distal heme pocket mutants: hydroxylation of 1-methoxynaphthalene

James E. Erman, Heather Kilheeney, Anil K. Bidwai, Caitlan E. Ayala, and Lidia B. Vitello

Department of Chemistry and Biochemistry, Northern Illinois University, DeKalb, IL 60115

Spectra of Russig's Blue in Organic and Aqueous Solvents

The spectra of Russig's blue in methylethylketone and in an aqueous phosphate buffer are shown in Figure A1.

Spectroscopic Scans of Russig's Blue Formation Catalyzed by CcP(triLeu), CcP(triVal) and rCcP

The spectroscopic changes associated with the CcP(triLeu)-catalyzed oxidation of 1-methoxynaphthalene by hydrogen peroxide to form Russig's blue are shown in Figure A2. Equivalent scans for the CcP(triVal)- and rCcP-catalyzed reactions are shown in Figures A3 and A4, respectively.

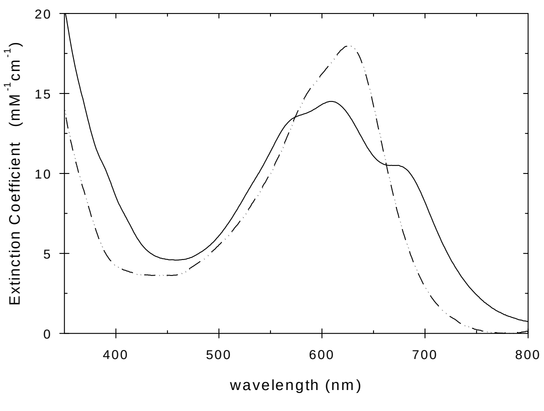

Fig. A.1. Spectra of Russig's blue in methylethylketone (dashed-dotted line) and in 0.100 M ionic strength, potassium phosphate buffer, pH 7.0 (solid line).

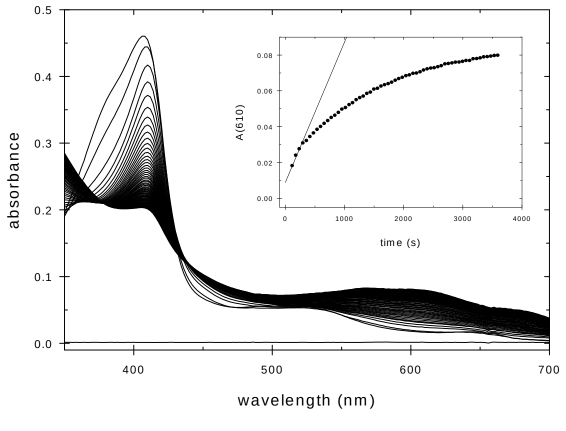

Fig. A.2. CcP(triLeu)-catalyzed oxidation of 1-methoxynaphthalene by H_2O_2. Spectra were collected every minute for one hour after addition of hydrogen peroxide. The large increase in absorbance between 450 and 800 nm is due to Russig's blue formation. Inset – Absorbance at 610 nm as a function of time. Experimental Conditions: [CcP(triLeu)] = 3.98 µM, [1-methoxynaphthalene] = 0.50 mM, $[H_2O_2]$ = 1.00 mM, pH 7.0, 25 °C.

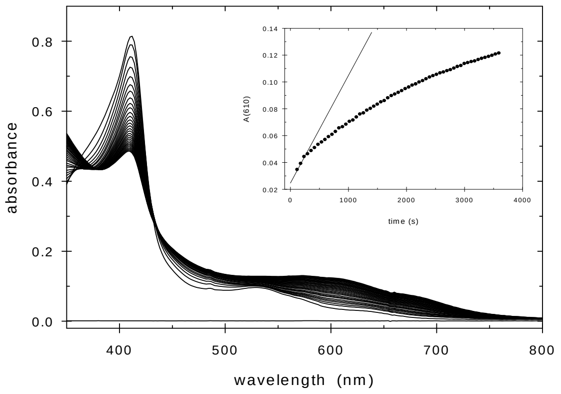

Fig. A.3. CcP(triVal)-catalyzed oxidation of 1-methoxynaphthalene by H_2O_2. Spectra were collected every minute for one hour after addition of hydrogen peroxide. The increase in absorbance between 450 and 800 nm is due to Russig's blue formation. Inset – Absorbance at 610 nm as a function of time. Experimental Conditions: [CcP(triVal)] = 10.2 µM, [1-methoxynaphthalene] = 0.50 mM, [H_2O_2] = 1.00 mM, pH 7.0, 25 ºC.

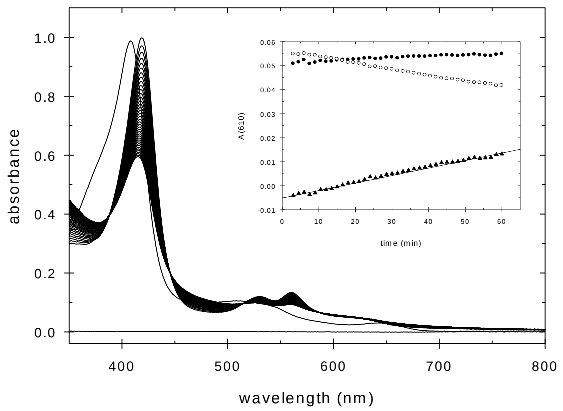

Fig. A.4. rCcP-catalyzed oxidation of 1-methoxy-naphthalene by H_2O_2. Spectra were collected every minute for one hour after addition of H_2O_2. Inset – Absorbance at 610 nm as a function of time in the presence (solid circles) and absence (open circles) of 1-methoxynaphthalene. Experimental Conditions: [rCcP] = 10.0 µM, [1-methoxynaphthalene] = 0.50 mM, [H_2O_2] = 1.00 mM, pH 7.0, 25 ºC.

Dependence of the Initial Velocity on 1-Methoxynaphthalene and Hydrogen Peroxide

The CcP(triAla)-catalyzed oxidation of 1-methoxynaphthalene depends upon both the substrate concentration, Figure A5, and the hydrogen peroxide concentration, Figure A6.

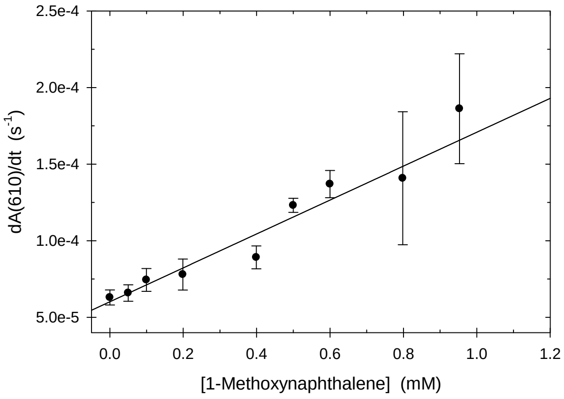

Fig. A.5. CcP(triAla)-catalyzed oxidation of 1-methoxynaphthalene by H_2O_2. The initial rate Russig's blue formation (monitored by the change in absorbance at 610 nm) as a function of the 1-methoxynaphthalene concentration. Experimental Conditions: [CcP(triAla] = 10 µM, [H_2O_2] = 1.00 mM, pH 7.0, 25 ºC.

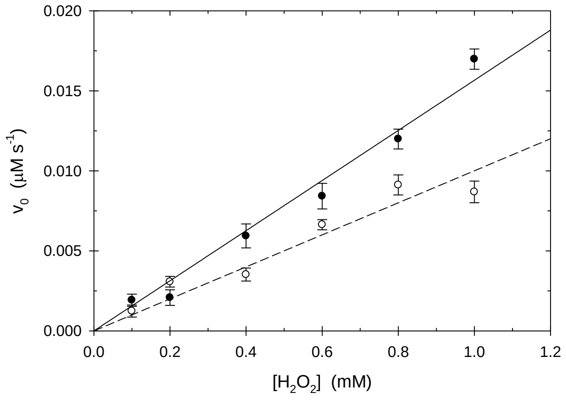

Fig. A.6. CcP(triAla)-catalyzed oxidation of 1-methoxynaphthalene by H_2O_2. The initial rate Russig's blue formation (monitored by the change in absorbance at 610 nm) as a function of the hydrogen peroxide concentration (filled circles). Rate of endogenous reduction of Compound I in the absence of 1-methoxynaphthalene (open circles).

Screening of CcP, CcP Mutants, and Other Heme Proteins for 1-Methoxynaphtalene Hydroxylation Activity and Heme Degradation

We screened all of the CcP heme pocket mutants that were available in our laboratory for 1-methoxynaphthalene activity to determine if any had unusually high peroxygenase activity. In addition to CcP(triAla), CcP(triVal), and CcP(triLeu), 17 other CcP heme pocket mutants were available. The turnover numbers under our standard reaction conditions are included in Figure A7 and Table A1. We have included the results of Shoji *et al.* [12] for cytochrome $P450_{BS\beta}$, HRP, and myoglobin and several myoglobin mutants in Table A1 for comparison. Cytochrome $P450_{BS\beta}$ has the highest activity by far, some three-orders of magnitude faster than any of the CcP variants. Since a myoglobin mutant, Mb(H64D), also had a very high peroxygenase activity we decided to include several other non-enzymatic heme proteins in the screening. These included *Glycera dibranchiata* hemoglobin (Glycera Hb), the heme domain of the direct oxygen sensor in *Escherichia coli* (EcDOSH), and the heme domains of FixL from *Bradyrhizobium japonicum* (BjFixLH) and from *Sinorhizobium meliloti* (SmFixLH).

We have used the decrease in Soret absorbance during the peroxygenase reaction as a measure of heme degradation during the reaction and these data are shown in Figure A8. CcP(H52N) has the lowest heme degradation while Glycera Hb has the highest. CcP(W51H) is the most sensitive CcP mutant in terms of heme degradation with CcP(triAla) second.

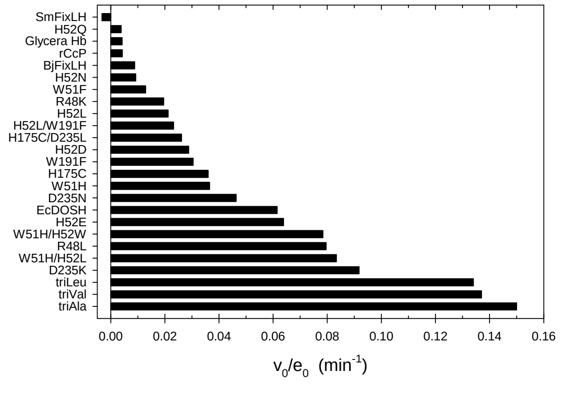

Fig. A.7. Turnover rates, v_0/e_0, for the hydroxylation of 1-methoxynaphthalene by hydrogen peroxide catalyzed by rCcP, 20 CcP mutants, and selected other heme proteins. Experimental Conditions: [1-methoxynaphthalene] = 0.50 mM, $[H_2O_2]$ = 1.00 mM, pH 7.0, 0.100 M ionic strength potassium phosphate buffer, 25 °C.

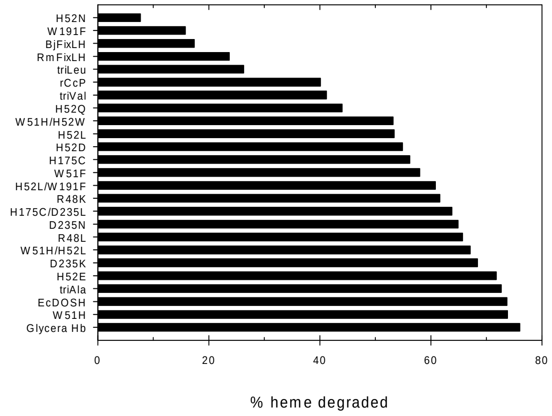

Fig. A.8. Heme degradation, as measured by percent decrease in the Soret absorbance after 30 minute exposure to 1.0 mM H_2O_2. CcP(H52N) shows the least heme degradation (8%) while *Glycera* Hb shows the greatest (76%). Experimental Conditions: pH 7.0, 25 °C.

Table A.1. Russig's Blue Activity of CcP, CcP Mutants, and Selected Heme Proteins.[a]

Enzyme	TN (min^{-1})	Enzyme	TN (min^{-1})
rCcP	0.0044 ± 0.0003	CcP(D235K)	0.092 ± 0.004
CcP(triAla)	0.150 ± 0.008	CcP(W51H/H52L)	0.083 ± 0.006
CcP(triVal)	>0.137	CcP(W51H/H52W)	0.078 ± 0.006
CcP(triLeu)	0.134 ± 0.014	CcP(H52L/W191F)	0.023 ± 0.001
CcP(R48L)	0.080 ± 0.002	CcP(H175C/D235L)	0.026 ± 0.001
CcP(R48K)	0.020 ± 0.001	Glycera Hb	0.0042 ± 0.0002
CcP(W51F)	0.013 ± 0.001	EcDOSH	0.062 ± 0.002
CcP(W51H)	0.037 ± 0.003	SmFixLH	-0.0033 ± 0.0002
CcP(H52D)	0.029 ± 0.002	BjFixLH	0.0089 ± 0.0002
CcP(H52E)	0.064 ± 0.002		
CcP(H52L)	0.021 ± 0.001	$P450_{BS\beta}$ [b]	112 ± 2
CcP(H52N)	0.0092 ± 0.0014	HRP [b]	ND [c]
CcP(H52Q)	0.0038 ± 0.0007	Mb [b]	0.03
CcP(H175C)	0.036 ± 0.001	Mb(H64A) [b]	1.1
CcP(W191F)	0.031 ± 0.002	Mb(H64D)[b]	23
CcP(D235N)	0.047 ± 0.002	Mb(F43H/H64L) [b]	1.5

[a] Experimental conditions: [1-methoxynaphthalene] = 0.50 mM, [H_2O_2] = 1.0 mM, pH 7.0, 0.10 M ionic strength, potassium phosphate buffer, 25 ºC. [b] ref. [12]. [c] ND = not detected
